# Enhancing Nature Connection and Positive Affect in Children through Mindful Engagement with Natural Environments

**DOI:** 10.3390/ijerph18094785

**Published:** 2021-04-30

**Authors:** Alexia Barrable, David Booth, Dylan Adams, Gary Beauchamp

**Affiliations:** 1 School of Education and Social Work, University of Dundee, Scotland DD1 4HN, UK; 2 School of Life Sciences, University of Dundee, Scotland DD1 4HN, UK; d.z.booth@dundee.ac.uk; 3 Cardiff School of Education, Cardiff Metropolitan University, Wales CF5 2XJ, UK; dadams@cardiffmet.ac.uk (D.A.); gbeauchamp@cardiffmet.ac.uk (G.B.)

**Keywords:** nature connection, children, nature reserves, affective wellbeing, mindfulness

## Abstract

Nature connection, which describes a positive relationship between humans and the rest of nature, has been recognised as a worthwhile goal of all education. Given its association with wellbeing, as well as the fact that it can predict ecological behaviours in children, there have been several calls for it to become central to environmental education, and an important tool in tackling climate change. Previous research has reported the success of short-term interventions in increasing nature connection in children, but to date no empirical studies have looked at how mindful engagement with nature can promote both nature connection and positive affect. This study took place in a nature reserve in Wales and included *n* = 74 children, aged 9–10, who took part in three mindful activities. Pre- and post- measures included nature connection and positive/negative affect. Analysis showed a significant small to medium effect of the activity on nature connection. Moreover, positive affect significantly increased post-activity, while negative affect showed a small decrease.

## 1. Introduction

There have been several calls in the last two decades for children to (re)connect with the natural world [1,2,3,4]. The reasons behind these calls are usually centred around two core aspects of nature connection: its positive association with increased wellbeing [5,6]; and pro-environmental attitudes and behaviours [7,8,9]. Formal education at all levels has been recognised as an avenue for promoting closer relationships with the natural world [10,11], with environmental education being well-placed to nurture such a relationship [8,12].

It is now generally recognised that nature contact and connection have many physical and psychological benefits. The relationship between nature connection and wellbeing has been studied widely, with two meta-analyses seeing a small to medium association [5,6]. A meta-analysis of the effect of exposure to nature on positive and negative affect showed a moderate increase in positive affect (PA) and a smaller decrease in negative affect (NA) [13].

In children, nature connection has been found to positively correlate with health outcomes and life satisfaction, albeit weakly, r = 0.09 and r = 0.14, respectively [14]. Positive correlations were also observed between connection to nature and time spent outdoors and in nature. It is notable that, in that study, the strongest correlations with nature connection were recorded with pro-environmental behaviours (r = 0.60). In another study with children, nature connection was found to be one of the main predictors of ecological behaviours in children [15].

A recent review of interventions to promote nature connection in children found fourteen studies that used one of the many validated measures of nature connection in pre- and post-testing [12]. The settings, activities and duration of the interventions described in these studies were extremely variable. Settings ranged from the South African bush to the Scottish Highlands [16] and from rainforests [17] to urban nature [18]. They also included indoor settings such as a natural history museum [18]. Activities included holiday camps [19], environmental education field trips with a knowledge basis [20], engagement with digital devices, i.e., Geogames [21] and sporting activities, such as surfing [22]. Finally, duration was variable, and ranged from a few hours to several weeks. Overall, many programmes showed positive changes in connection to nature, with only a few, however, recording a sustained change at follow up [17,23] with younger age groups (below 11) having a higher likelihood of maintaining higher levels of nature connection.

Research in adults suggests that there are certain types of activities that promote connection to nature more readily than others. For example, engaging with nature’s beauty, and feeling positive emotions and empathy towards the natural world are more likely to promote a positive affective relationship [24]. Similarly, there are several studies in adults that suggest that paying mindful attention or undertaking mindful engagement with the natural world [12,25,26] leads to substantial increases in nature connection even when the interventions undertaken were short. Moreover, other research, also in adults, has noted that mindfulness can not only enhance our connection to nature, but it can also decrease negative affect [27]. It is interesting to note that the mindful experience in nature did not increase positive affect (PA) in this study. Sato, Jose and Conner [28] report on a study that looked at the mediating effect of ‘savouring’ of nature on PA. The characteristics of ‘savouring’, such as the element of being absorbed in the experience, and the sensory-perceptual sharpening as recognised by Sato et al. [28], are not dissimilar to the experience of mindful attention [29]. There is, therefore, potential to explore how mindful engagement with the natural world may present an important pathway in building a positive relationship with nature. The role of positive affect in that is also of interest.

The construct of nature connection has many aspects to it, including an affective one. Our feelings and emotions towards the natural world inform how we see our relationship to it, and whether we consider ourselves a part of it. Affective outcomes of outdoor education programmes have been widely recognised as desirable and important [30] Such a focus may co-exist with other outcomes, such as knowledge, behaviour and attitude [31] although there is some evidence to suggest that high information content may have a negative effect on the development of positive affective outcomes [32].

To the best of our knowledge, there have been no previous studies of a quantitative nature in children that specifically look at mindful engagement with nature as a potential pathway to nature connection, though in some sense other types of activities may promote such engagement (for example nature photography in Bruni) [18]. Moreover, and to the best of our knowledge, no previous research has looked at the association between nature connection and affective wellbeing in children.

## 2. The Current Study

The current study aims to examine the changes in nature connection and affect in 9–10-year-old primary school pupils after participation in a field trip at a nature reserve. According to previous research, this age group is more likely to show a marked increase in nature connection after an intervention, and sustain this change in follow up [12]. The activities that the children undertook were designed to give the participants a mindful engagement with the natural world and are described below. The following hypotheses were put forward:

(1) Children taking part in the mindful engagement with nature activity will show an increase in their nature connection scores.
(2) Children taking part in the mindful engagement with nature activity will show an increase in their positive affect immediately after the activity.
(3) Children taking part in the mindful engagement with nature activity will show a decrease in their negative affect immediately after the activity.
(4) Changes in nature connection will persist upon follow up eight weeks after the activity.

## 3. Method

### 3.1. Participants

This paper reports on the results of pre- and post-activity questionnaires with children aged 9–10 years, who took part in outdoor activities at a nature reserve. Overall, four classes from four separate Welsh state primary schools (A, B, C and D) took part in the activity. This was a convenience sample, of schools which have an ongoing relationship with the university and researcher undertaking the project, as well as parents and children who agreed to participate in this research project. The overall number of participants was *n* = 97, 33 of whom were female, 29 were male and 12 had no data on sex. As 23 participants with missing, incomplete or unpaired observations were omitted from further analysis, the total number was analysed at *n* = 74. The age range of the children was 9–10 with a mean of 9.51 years of age.

Ethical approval was sought from the university ethics committee before beginning research. Headteachers and teachers, who can be seen as the “gatekeepers” of access to the school and children also gave their approval. Children had a live presentation by the lead researcher to explain the process and activities prior to taking a letter home in order to gain consent from their parents or guardians. Each child was also given a letter of assent in order to ensure that they could decide whether to take part or not. In line with BERA (2011) guidelines, children were given the opportunity to ask questions and were clearly informed of their right to withdraw “for any or no reason, and at any time” [33].

### 3.2. Procedure

The children participating in the project completed the initial questionnaire in their classroom. Two days later, the children visited the nature reserve and undertook three mindfulness-based activities designed to enhance mindful engagement with nature. The definition of mindfulness is contested; however, Bishop et al. [34] outline two common components of mindfulness. First, they state that mindfulness involves “the self-regulation of attention so that it is maintained on immediate experience, thereby allowing for increased recognition of mental events in the present moment” [34]. The second common component is that it “involves adopting a particular orientation toward one’s experiences in the present moment, an orientation that is characterized by curiosity, openness, and acceptance” [34]. The activities were chosen as it was felt that they were able to achieve these two components. The first mindfulness activity involved mindful listening to nature sounds and the second mindfulness activity involved mindful looking at nature near and far, such as flowers and mountains. The third activity was a pretend hunting game. During this game the children pretended to be animals. One group was the hunting animal (hyena) and the other was the hunted (deer). The roles were swapped, so all children experienced both aspects of the game. Schools A, C and D had all the experiences in the planned timescale of two and a half hours. However, due to inclement weather, school B’s activities were shorter in duration by approximately 30 min in total.

After undertaking the activities and returning to school, all children completed the second questionnaire in their classrooms. Finally, some of the pupils completed the follow-up questionnaire eight weeks post-activity. As schools were closed at the time due to the COVID-19 pandemic, researchers were unable to contact all children who had taken part. This is addressed in the analysis.

### 3.3. Measures

The following measures were used. All children completed the pre- and post-activity questionnaire that included two nature connection measures, namely the Nature Connection Index (NCI) [35] and the Inclusion of Nature in Self (INS) [36]. Additionally, the questionnaire included a measure of affect, namely, the Positive Affect, Negative Affect Scale for Children (PANAS-C) [37], as well as simple demographic characteristics, such as participant age and sex.

### 3.4. Nature Connection Index

This is a recently developed measure, validated for the use of children as young as seven years of age, as well as adults. One of the reasons that this particular measure was used is that it has previously been shown to have no ceiling effect, something that has been noted as a limitation in previous measures used in similar interventions [12]. Moreover, the NCI has shown adequate sensitivity to measure changes in applied interventions such as this one [35]. It is a six-item, seven-Likert response scale that unlike other, longer measures, focuses mainly on affective and experiential as opposed to cognitive factors. Sample items include statements such as “I always find beauty in nature” and “I find being in nature really amazing”, while the responses range from “completely disagree” to “completely agree”. In terms of internal consistency, the original development and validation study reports a Cronbach’s alpha of α = 0.92 [35], while in this study the alpha was calculated at α = 0.87 ± 0.05.

### 3.5. Inclusion of Nature in Self

The INS [36] is a pictorial scale for use with children and adults. It consists of seven progressively more interconnected circles, one of which represents the “self”, and the other “nature”. It has previously been used with children as young as eight years of age [38]. As the INS is a single item measure, no internal validity scores are calculated, but a previous study [39] reports good convergent validity with other nature connection measures. In this study, the correlation between the NCI and the INS was r = 0.59.

### 3.6. Positive Affect, Negative Affect Scale for Children 

The PANAS-C [37] is a 10-item, five-Likert response scale that measures the self-reported frequency of positive and negative affects in children from the age of eight upwards. It includes both positive and negative affect words, with sample items for positive affect being “lively” and “joyful” and negative affect “sad” and “scared”. In this instance, the original word “mad” was changed to “angry” to reflect British English usage. The internal validity of the PANAS-C in the original validation study was reported as α = 0.88. While in this study the alpha was calculated at α = 0.87 ± 0.05.

## 4. Analysis

All graphing and analyses were conducted in the R environment [40]. Survey results were collated in Microsoft Excel (version 365). Missing and incomplete data, including partially completed surveys, i.e., unmatchable pre/post responses, no participant ID and partial responses to NCI or PANAS-C, were not included in the analysis.

Overall NCI scores were calculated as the sum of the six-question survey, weighted [35]; the PA and NA scales were calculated as the unweighted sum of five questions within the ten-question PANAS survey [41]. The Inclusion of Nature in Self scale [36] was encoded as letters A–G, and converted to a numeric scale of 1–7 for correlation. These instruments were correlated and bootstrapped with 1000 repeats.

A follow-up questionnaire was completed eight weeks later; however, only twenty participants from two of the schools were able to participate with only a subset in a complete state for analysis (*n* = 10); as such these results are descriptively reported below (see supplementary Table S2) with inferential statistical analysis deemed inappropriate.

Statistical analyses were conducted for the three instrument scores against predictors. As the NCI, PA (positive affect) and NA (negative affect) generate proportional-scored responses, a mixed effect generalised linear model (GLM) with a binomial error distribution was fitted to the predictor variables using the lme4 [42] as the scores are bounded and proportional in nature. To achieve this, proportional scores were fitted using a logit link by maximum likelihood (Laplace approximation) to fit the following set of linear terms with School, Timing of survey (fixed effects), and Participant (random effect) in the formula to produce a saturated model.

y~School + Timing + School: Timing + (1|Participant), family = “binomial”(1)

This saturated model was simplified using backward elimination with a likelihood ratio (LR) test at each step to assess goodness of fit, and to justify the removal of a predictor to the minimal adequate model. This model was finally contrasted against a null model (a model with only intercepts). The formula for the LR test statistic is as follows:

LR = −2ln((L(simplified))/(L(complex))) = 2(loglik(complex) − loglik(simplified))(2)

The minimal adequate model was bootstrapped with 1000 replicates to estimate a 95% confidence interval on the regression coefficients. As three models were fitted to these data, a Bonferroni correction was applied to account for family-wise error (adjusted *p*-value threshold of 0.017). As a complimentary conservative approach in order to adjust for false discovery rate, the Benjamini and Hochberg approach was used [43]; these were found to produce identical adjustments. Only coefficients that remained significant after adjustment are presented.

PANAS-C scores were decomposed to their individual affect scores with a paired Wilcoxson signed rank test conducted to determine change between the pre and post condition. To counteract familywise error, *p*-values were adjusted with a Bonferroni correction.

## 5. Results

**Figure 1:**
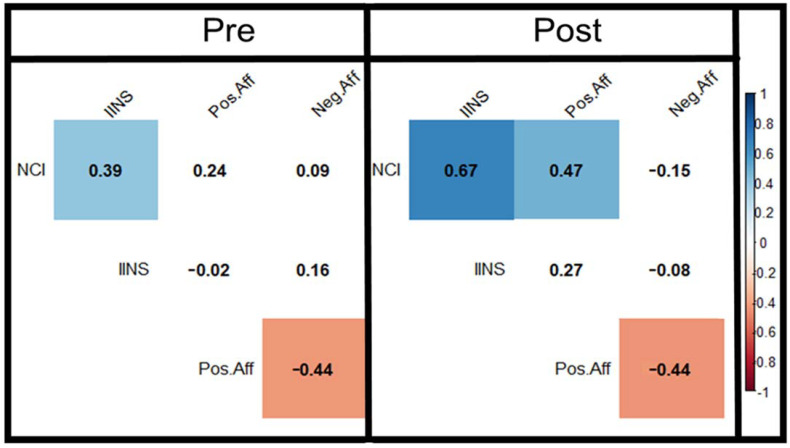
Pre and post correlation coefficients for the NCI, IINS and PANAS-C instruments. Non-significant correlation values given with white colouring. Blue indicates positive correlation; red indicates negative.

Correlation of the instruments in the study are presented in Figure 1 (and Supplementary Table S1), with NCI and INS and NCI and PANAS-C positive affect having significant positive correlations post intervention. PANAS-C negative affect was found to only have a significant negative correlation with the PANAS-C positive affect score in both the pre and post conditions.

Descriptive results are presented in Table 1. Scores for the NCI instrument spanned the full range of the scale (NCI 9–100), whereas the PANAS-C scores were truncated (PANAS-C positive affect 8–25; PANAS-C negative affect 5–15). NCI and PANAS-C positive affect increased between the pre- and post-activity survey, whereas PANAS-C negative affect slightly decreased. These shifts were reflected in the mean values overall and the differential calculated for the participants (see Table 1).

Minimal adequate regression models are presented in Table 2. With respect to the NCI, this shift due to the timing of the survey was observed to be a small- to medium-sized effect (McFadden’s pseudo R-squared = 0.23). With PANAS, there was a small- to medium-sized effect (McFadden’s pseudo R-squared = 0.13) increase in positive affect, which was observed post activity; finally a small-sized effect of a drop in negative affect effect (McFadden’s pseudo R-squared = 0.02) was observed post activity.

**Figure 2:**
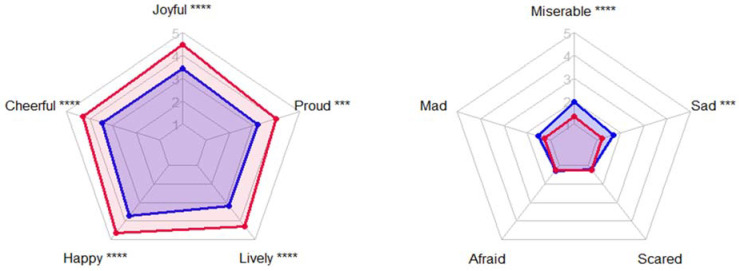
Means of the PANAS-C affect in the pre and post timing conditions. Pre-blue polygon, Post-red polygon. *** *p* ≤ 0.001; **** *p* ≤ 0.0001.

Decomposing the PANAS-C into individual questions, it was found that all the positive affect questions increased after the activity, with “Joyful”, “Happy” and “Lively” having the largest magnitude change. Within the negative affect set only “Miserable” and “Sad” were found to decrease significantly (see Table 3 and Figure 2).

## 6. Discussion

This study examined the changes in nature connection and affect in 9–10-year-old primary school pupils after participation in a field trip at a nature reserve. In terms of nature connection, we observed an upwards shift between the pre- and post-scores, of a small to medium effect size. This is not dissimilar to other studies that have looked at short-term nature interventions in children of this age. Effect sizes equivalent in magnitude have been observed with other longer interventions, such as wildlife expeditions [16], summer camps [19] and in one-day environmental education programs [32,44]. It could be argued that the time spent in nature, rather than the mindfulness activities, was the cause of changes in nature connection affect. However, all the schools regularly undertake prolonged time in nature in their school grounds as part of “Forest School” session. Moreover, previous theoretical work suggests that it is not contact alone that promotes connection to nature, but rather that the quality of the interaction matters, with mindfulness being recognised as one of the pathways [24,45].

It is worth mentioning that there was some heterogeneity observed between the schools (the separate results can be seen in supplemental Table S2). The post-activity nature connection score of schools A, C and D showed a significant increase. School B, who had the shorter activities, did not have the same increase in nature connection. This may point towards a negative impact of certain weather conditions on how we feel about the natural world around us, and about our connection to it, and could merit further research.

In terms of changes in affect, all children in all groups showed a small- to medium-sized effect in the increase in PA. Exploratory analysis showed that all of the positive emotions saw significant change between the pre-activity scores and the post-activity scores, while only ‘miserable’ and ‘sad’ had a significant change in terms of NA. Further qualitative investigation of the emotions experienced during the activity, which has been undertaken, will probably give a clearer idea of the ways in which a mindful engagement with nature can promote PA, and reduce NA.

These results support Bonnett’s [46] claim that “if we enter a natural place and participate in the vibrancy of its being our own embodied being is enlivened and refreshed” (p. 339) and this enables our “ecstatic nature” to be fulfilled. In other words, our instinctive and authentic sense of being is bound up in our experience of nature. Bonnett [46] also argues that, if children are allowed to participate with the otherness of nature, then this can lead to augmented existential understandings, an enhanced sense of wellbeing, and can enable human flourishing.

This research study has shown that children’s affinity with the natural world can be nurtured through mindfulness activities in nature reserves. It is hoped that this affinity may translate into an improved willingness from the participating children to take care of the natural world as “simply put, humans don’t protect what they don’t know and value” [47]. As Sobel [48] explains, “if we want children to flourish to become truly empowered let us allow them to love the earth before we ask them to save it”. Further research could explore this area and also address, in more detail, the qualitative dimension of children’s sense of connectivity to nature.

These results can be seen to be educationally positive if children’s wellbeing, their understandings of their feelings, identity, and increased connection with nature are viewed as educationally desirable. It is argued, however, that these concerns are not congruent with the current curricula of mainstream education, both in the UK and internationally. For instance, Pulkki et al [49] argue that even environmental education curricula neglect focusing on students’ embodied experiences. Consequently, the school curriculum is “almost never about what goes on ‘in me’ and in my lived- body” and therefore, “the hidden curriculum of all mainstream education is that you, your thoughts/feelings/experiences, do not really matter” [49]. Instead, rational knowledge is prioritised over biophilic, mindful experiences that could connect students to nature. They claim that this lack of understanding is “perpetuated by modern developments like globalisation, industrialisation, rationalisation and commercialisation” [49] as they hinder our awareness of place and our body’s connection with the natural world. In so doing, they “alienate us from the very nutrients that sustain our lives” [49].

## 7. Limitations

There are a series of limitations of this research, some focusing on research design. As with many of the research projects performed within educational institutions, there was no control group and no opportunity for randomisation. One of the ways that we could have overcome that was a wait-control group but, in this particular instance, this was not possible due the timing of the trips and class time availability. We are, therefore, limited in our interpretation of these results. Another limitation of this project is the lack of follow up. Barrable et al. [12], in their recommendations for more robust research in interventions of nature connection, mention the taking of follow-up measures as good practice. In this instance, a follow up at eight weeks post-activity had been planned but due to the closing of schools because of the COVID-19 pandemic, such a follow up was only partially undertaken. The raw numbers are presented in Supplementary Table S2.

## 8. Conclusions

Finding ways to nurture the connection that children have with the non-human natural world holds the promise of supporting wellbeing and mental health, as well as increasing pro-environmental attitudes and behaviours. As is highlighted above, many highlight the need to nurture a love of nature through direct experiences. Children need to feel empathy and care before they are told that they need to care, if sustainable environmental behaviours are to be achieved [48]. Sobel states that “the malaise of ecophobia” can be cured “with ecophilia-supporting children’s biological tendency to bond with the natural world”. In this study we showed that even a short (half-day) intervention, such as this one that included a series of mindful engagements with the natural world, could be of benefit to children in supporting the building of a positive relationship to the natural world. Although it is not possible, due to methodological limitations to disentangle whether it was the mindfulness, or simply the present in nature reserves that is causal to the increase in nature connection, we can certainly say that such activities in nature reserves can have a positive effect on children connection with the natural world.

This positive relationship is important not just because it can promote wellbeing and could help cultivate pro-environmental attitudes, but also because it can provide enhanced existential understandings [46]. As Bonnett explains, mindful engagement with nature enables experiences of “ecstasis” that can re-open a sense of wonder and “otherness” that far outruns “any preconceptions and knowingness with which habitually we equip and insulate ourselves”. Overall, we suggest that our results should encourage educators to make use of local natural spaces, such as nature reserves, as well as parks and other green and blue spaces, both for educational as well as recreational activities on a regular basis. Such use, coupled with different types of activities that can help children engage mindfully, can help support the development of a healthy and positive relationship with the natural world, and support children’s affective and holistic wellbeing.

## Figures and Tables

**Table 1:** Sample size, mean, confidence interval and pre–post data for schools A–D.

Instrument		Pre		Post		Delta	
	*n*	$\overset{—}{x}$ [sd]	CI	$\overset{—}{x}$ [sd]	CI	$\overset{—}{x}$ [sd]	CI
**NCI**	74	44.87 [22.27]	[39.72–50.04]	59.84 [25.93]	[53.83–65.84]	15.27 [23.15]	[9.87–20.67]
**PANAS-C (PA)**	74	17.01 [4.91]	[15.88–18.15]	21.55 [3.94]	[20.64–22.47]	4.63 [4.87]	[3.49–5.77]
**PANAS-C (NA)**	74	7.74 [2.74]	[7.11–8.38]	6.35 [1.99]	[5.89–6.81]	−1.41 [2.91]	[−2.09–−0.73]

**Table 2:** Predictors of survey NCI, PANAS positive and negative affect from minimal adequate models after Bonferroni correction.

DV	Coefficients	B	*SE*	Z	*p*
**NCI**	Intercept	−0.7	0.25	−2.83	<0.01
	Timing	1.16	0.07	17.38	<0.001
	School B: Timing	−1.10	0.10	−11.15	<0.001
	School D: Timing	−0.49	0.10	−4.85	<0.001
		McFadden’s R-squared	0.23		
**PANAS Positive affect**	Intercept	0.27	0.07	3.77	<0.001
	Timing	0.71	0.07	10.79	<0.001
		McFadden’s R-squared	0.13		
**PANAS Negative affect**	Intercept	−1.07	0.05	−20.09	<0.001
	Timing	−0.26	0.07	−3.58	<0.001
		McFadden’s R-squared	0.02		

**Table 3:** Positive and negative affect components tested between pre and post survey timing.

Affect	Question	95% CI of Difference	*V*	*p-val*	*sig*
Positive	Joyful	[1.50 2.00]	1246	2.60 × 10^−8^	****
	Cheerful	[1.00 1.50]	985	5.18 × 10^−6^	****
	Happy	[1.00 2.00]	906	1.00 × 10^−7^	****
	Lively	[1.00 2.50]	1013	1.70 × 10^−6^	****
	Proud	[0.50 2.00]	926	4.50 × 10^−4^	***
Negative	Miserable	[−1.50 −1.00]	117	9.13 × 10^−6^	****
	Angry	[−2.00 0.00]	77	0.032	NS
	Afraid	[−0.50 1.00]	129	0.96	NS
	Scared	[−1.00 1.00]	132	0.57	NS
	Sad	[−2.00 −0.50]	104	1.30 × 10^−3^	**

NS not significant; ** *p* ≤ 0.01; *** *p* ≤ 0.001; **** *p* ≤ 0.0001.

## Supplementary Materials

The following are available online at https://www.mdpi.com/article/10.3390/ijerph18094785/s1. Table S1: Instrument correlations (lower matrix) and confidence intervals (upper matrix) pre and post. Table S2: Descriptive statistics by School. Figure S1: (A) Positive correlation of NCI with IINS; (B–D) Interaction plot of NCI, PANAS positive and negative affect as a function of questionnaire.
