# Supplementary material for: Enhancing Nature Connection and Positive Affect in Children through Mindful Engagement with Natural Environments
Source: Int J Environ Res Public Health. 2021 Apr 30;18(9):4785. doi: 10.3390/ijerph18094785 (PMC8125517; doi:10.3390/ijerph18094785)
Supplement: Supplementary file 1 [file ijerph-18-04785-s001.zip › ijerph-1159102-supplementary.pdf]

Table S1. Instrument correlations (lower matrix) and confidence intervals (upper matrix) pre and post.

**Pre**	**NCI**	**IINS**	**Pos. Aff**	**Neg. Aff**
NCI		[0.19–0.58]	[0.00–0.46]	[−0.16–0.35]
IINS	0.39		[−0.26–0.21]	[−0.06–0.37]
Pos. Aff	0.24	−0.02		[−0.58–−0.28]
Neg. Aff	0.09	0.16	−0.44	
**Post**	**NCI**	**IINS**	**Pos. Aff**	**Neg. Aff**
NCI		[0.53–0.81]	[0.31–0.64]	[−0.35–0.05]
IINS	0.7		[0.03–0.52]	[−0.30–0.13]
Pos. Aff	0.5	0.27		[−0.66–−0.23]
Neg. Aff	0	−0.08	−0.44	

Table S2. Descriptive statistics by School.

			Pre		Post			Delta	
Instrument	School	*n*	$\bar{x}$ [sd]	CI	$\bar{x}$ [sd]	CI		$\bar{x}$ [sd]	CI
NCI	A	23	35.26 [17.82]	[27.55 - 42.97]	59.61 [24.92]	[48.83 - 70.39]		24.35 [23.57]	[14.16 - 34.54]
	B	19	48.84 [21.62]	[38.42 - 59.26]	50.11 [26.18]	[37.49 - 62.72]		1.26 [14.25]	[-5.61 - 8.13]
	C	15	49.33 [27.72]	[33.98 - 64.69]	68.47 [26.61]	[53.73 - 83.2]		19.13 [23.37]	[6.19 - 32.07]
	D	17	49.53 [20.86]	[38.8 - 60.25]	63.41 [25.02]	[50.55 - 76.27]		13.88 [24.64]	[1.21 - 26.55]
PANAS Positive Affect	A	23	16.39 [4.08]	[14.63 - 18.15]	21.43 [4.4]	[19.53 - 23.34]		5.04 [5.12]	[2.83 - 7.26]
	B	19	16.68 [4.52]	[14.50 - 18.86]	20.53 [3.42]	[18.88 - 22.18]		3.84 [4.1]	[1.87 - 5.82]
	C	15	16.21 [6.34]	[12.55 - 19.87]	22.53 [3.56]	[20.56 - 24.51]		6.86 [5.92]	[3.58 - 10.14]
	D	17	18.88 [5.01]	[16.31 - 21.46]	22.00 [4.18]	[19.85 - 24.15]		3.12 [3.92]	[1.1 - 5.13]
PANAS Negative affect	A	23	7.22 [2.68]	[6.06 - 8.38]	6.26 [2.28]	[5.27 - 7.25]		-.96 [3.28]	[-2.38 - .46]
	B	19	8.74 [2.81]	[7.38 - 10.09]	6.63 [2.19]	[5.58 - 7.69]		-2.11 [3.31]	[-3.7 - -.51]
	C	15	7.93 [2.46]	[6.57 - 9.3]	6.73 [2.09]	[5.58 - 7.89]		-1.2 [1.82]	[-2.21 - -.19]
	D	17	7.18 [2.86]	[5.71 - 8.64]	5.82 [1.13]	[5.24 - 6.41]		-1.35 [2.71]	[-2.75 - .04]

			Follow-up	
Instrument	School	*n*	$\bar{x}$ [sd]	CI
NCI	B	10	31.5 [6.28]	[27.01 - 35.98]
	C	10	35.1 [5.85]	[30.91 - 39.29]
PANAS Positive Affect	B	10	17.8 [6.03]	[13.48 - 22.12]
	C	10	18.9 [5.93]	[14.65 - 23.14]
PANAS Negative affect	B	10	8.6 [3.24]	[6.28 - 10.92]
	C	10	8.8 [4.76]	[5.4 - 12.2]

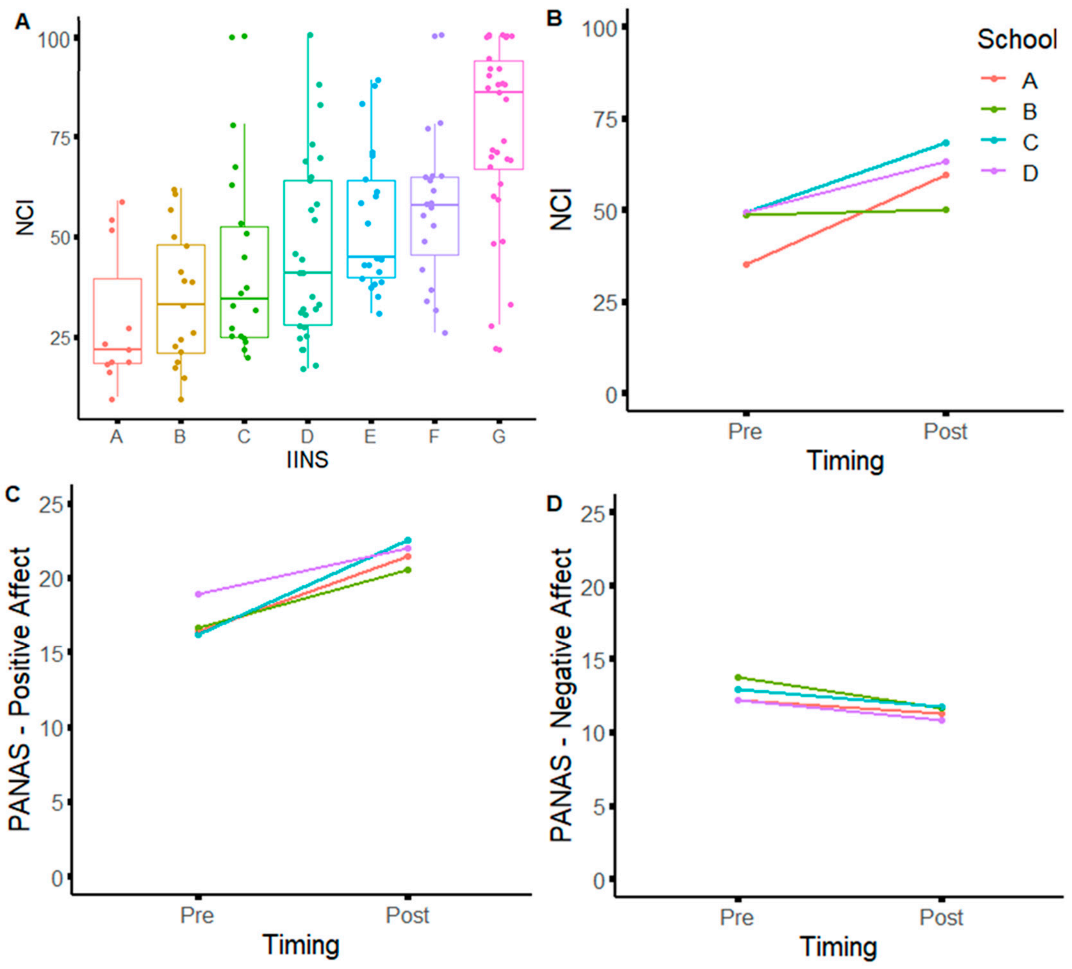

Figure S1. (**A**) Positive correlation of NCI with IINS; (**B**–**D**) Interaction plot of NCI, PANAS positive and negative affect as a function of questionnaire.
